# Enhancing Functional Recovery Through Intralesional Application of Extracellular Vesicles in a Rat Model of Traumatic Spinal Cord Injury

**DOI:** 10.3389/fncel.2021.795008

**Published:** 2022-01-03

**Authors:** Pasquale Romanelli, Lara Bieler, Patrick Heimel, Siniša Škokić, Dominika Jakubecova, Christina Kreutzer, Pia Zaunmair, Tomislav Smolčić, Bruno Benedetti, Eva Rohde, Mario Gimona, David Hercher, Marina Dobrivojević Radmilović, Sebastien Couillard-Despres

**Affiliations:** ^1^Institute of Experimental Neuroregeneration, Spinal Cord Injury & Tissue Regeneration Centre Salzburg (SCI-TReCS), Paracelsus Medical University, Salzburg, Austria; ^2^Innovacell AG, Innsbruck, Austria; ^3^Core Facility Hard Tissue and Biomaterial Research, Karl Donath Laboratory, University Clinic of Dentistry, Medical University Vienna, Vienna, Austria; ^4^Ludwig Boltzmann Institute for Traumatology, The Research Center in Cooperation with AUVA, Vienna, Austria; ^5^Austrian Cluster for Tissue Regeneration, Vienna, Austria; ^6^Croatian Institute for Brain Research, University of Zagreb School of Medicine, Zagreb, Croatia; ^7^GMP Unit, Spinal Cord Injury & Tissue Regeneration Centre Salzburg (SCI-TReCS), Paracelsus Medical University, Salzburg, Austria; ^8^Department of Transfusion Medicine, University Hospital, Salzburger Landeskliniken GesmbH (SALK) and Paracelsus Medical University, Salzburg, Austria; ^9^Transfer Centre for Extracellular Vesicle Theralytic Technologies (EV-TT), Salzburg, Austria; ^10^Research Program “Nanovesicular Therapies”, Paracelsus Medical University, Salzburg, Austria; ^11^Department of Histology and Embryology, University of Zagreb School of Medicine, Zagreb, Croatia

**Keywords:** exosomes, inflammation, motor function, locomotion, neuroregeneration, traumatic spinal cord injury, scarring, neuroimaging

## Abstract

Local inflammation plays a pivotal role in the process of secondary damage after spinal cord injury. We recently reported that acute intravenous application of extracellular vesicles (EVs) secreted by human umbilical cord mesenchymal stromal cells dampens the induction of inflammatory processes following traumatic spinal cord injury. However, systemic application of EVs is associated with delayed delivery to the site of injury and the necessity for high doses to reach therapeutic levels locally. To resolve these two constraints, we injected EVs directly at the lesion site acutely after spinal cord injury. We report here that intralesional application of EVs resulted in a more robust improvement of motor recovery, assessed with the BBB score and sub-score, as compared to the intravenous delivery. Moreover, the intralesional application was more potent in reducing inflammation and scarring after spinal cord injury than intravenous administration. Hence, the development of EV-based therapy for spinal cord injury should aim at an early application of vesicles close to the lesion.

## Introduction

Traumatic spinal cord injury (tSCI) is a complex clinical condition considered to be one of the most debilitating neurological disorders in industrialized societies. The initial tissue disruption following injury triggers an inflammatory response aggravating the spinal cord lesion (David and Kroner, 2015; Ahuja et al., 2017; Couillard-Despres et al., 2017). It is well documented that the upregulation of pro-inflammatory cytokines during the early phase of secondary damage leads to further neuronal death and motosensory losses (Pineau and Lacroix, 2007; Bastien and Lacroix, 2014; Anwar et al., 2016).

The principal sources of pro-inflammatory cytokines at the lesion site are the microglia and the “classically-activated” macrophages (Pineau and Lacroix, 2007; Zhou et al., 2014; Honjoh et al., 2019). The former are the resident immune cells of the central nervous tissue, which upregulate the secretion of pro-inflammatory cytokines, such as interleukin-1α and β (IL-1α and IL-1β), interleukin 6 (IL-6) and tumor necrosis factor-α (TNF-α), very rapidly after tSCI (Hayashi et al., 2000; Pan et al., 2002; Yang et al., 2004; Pineau and Lacroix, 2007). These cytokines are responsible for the recruitment of circulating leukocytes to the lesion site and for fostering their activation. Activated microglia and blood-derived macrophages maintain a deleterious inflammatory state leading, among other issues, to apoptosis of oligodendrocytes, activation of astrogliosis, and formation of a fibroglial scar (Fitch et al., 1999; Lu et al., 2000; Beattie et al., 2002; Gao et al., 2013; Ren and Young, 2013; Garcia et al., 2016). The Janus-faced nature of the fibroglial scar resulted in discrepant findings showing beneficial and deleterious properties. However, the scar is believed to impede or even block regenerative processes (Karimi-Abdolrezaee and Billakanti, 2012; Anderson et al., 2016; Bellver-Landete et al., 2019; Bradbury and Burnside, 2019).

Hence, the over-expression of pro-inflammatory cytokines initiates and sustains the relentless inflammatory response, which exacerbates tissue injury and hinders functional recovery (Popovich et al., 1997; Kigerl et al., 2009; Prüss et al., 2011; Ahuja et al., 2017). Studies on tSCI patients have shown a correlation during the acute stage post-injury between lower concentrations of pro-inflammatory cytokines (e.g., TNF-α and IL-1β) in the blood and CSF, and a favorable neurological outcome (Biglari et al., 2015; Kwon et al., 2017). This observation underscores the importance of controlling the inflammatory processes as early as possible after the initial trauma. Yet, although many “single-molecule” therapies have been explored for the treatment of tSCI, effective treatments improving functional recovery remain controversial in pre-clinical studies and represent an unmet medical need.

Using a rat tSCI contusion model, we recently demonstrated that an early intravenous application of human umbilical cord mesenchymal stromal cells (hUC-MSCs) reduced inflammation and astrogliosis at the site of injury (Romanelli et al., 2019). Our observation supports previous reports (Chen et al., 2008; Quertainmont et al., 2012; DePaul et al., 2015; Badner et al., 2016), which showed that the intravenous administration of MSCs decreased the inflammatory response after tSCI in rats. These reports also showed that MSCs application elevated serum levels of anti-inflammatory cytokine IL-10 and decreased levels of pro-inflammatory TNF-α. However, the unforeseen need for MSCs in an acute intervention following tSCI precludes the timely use of patients’ own MSCs, which would first need to be expanded *ex vivo*. Therefore, acute MSC-based therapies for tSCI are bound to be performed with allogenic material. In addition, the transplantation of proliferative whole cells constitutes a considerable risk for complications and tumorigenicity (Barkholt et al., 2013).

Accumulating evidence suggests that the biological and therapeutic effects of MSCs are contained in secreted factors acting over long distances, rather than resulting from direct cell-cell interactions within the lesioned tissues (Caplan and Dennis, 2006; Ruppert et al., 2018; Zhou et al., 2019). Among the various factors secreted by MSCs, extracellular vesicles (EVs) represent promising candidates to mediate the biological activities associated with MSCs and could provide a breakthrough for the development of novel therapies (Gimona et al., 2017; Campanella et al., 2019; Rohde et al., 2019). Through their capacity to transfer bio-active molecules, such as DNA, messenger RNAs (mRNAs), microRNAs (miRNAs), proteins, and lipids (Colombo et al., 2014), EVs are increasingly being recognized as important components of intercellular communication for numerous physiological and pathological processes. Moreover, EVs can reach distant targets and readily cross barriers, such as the blood-brain barrier (Saint-Pol et al., 2020).

We reported recently that an intravenous administration of hUC-MSCs-derived extracellular vesicles (named EVs hereafter) following tSCI reduced the inflammatory and scarring processes more efficiently than the application of their parental cells (Romanelli et al., 2019). In line with our findings, other studies have reported that the administration of MSC-derived EVs attenuated inflammation and improved functional recovery after tSCI (Huang et al., 2017; Ruppert et al., 2018; Liu et al., 2020; Noori et al., 2020). The possibility that a higher local concentration and a faster contact with the lesioned tissue further potentiate the beneficial activities of EVs following tSCI remained to be examined. In this study, we compared the long-term functional and structural outcomes obtained following acute intralesional or systemic application of EVs secreted by hUC-MSCs in a rat spinal contusion model.

## Materials and Methods

### Purification of EVs

Single donor-derived human umbilical cord multipotent stromal cells (hUC-MSC at passage number 4) were expanded in alpha-modified minimum essential medium (α-MEM, Sigma, Darmstadt, Germany) supplemented with 10% v/v pooled human platelet lysate (pHPL) and dipeptiven (5.5 mg/ml, Fresenius-Kabi, Graz, Austria). The growth medium was fibrin-depleted by centrifugation at 2,500× *g* for 20 min and further clarified by filtration through a 0.22 μm Stericup filter (Merck, Darmstadt, Germany). At 70% confluence, cells were washed twice with phosphate-buffered saline (PBS, Sigma) and the culture medium was replaced with pHPL-EV-depleted medium, which was prepared as described earlier (Pachler et al., 2017). After 24 h, EVs were isolated from 2.5 L of conditioned medium (CM) derived from 336 × 10^6^ hUC-MSCs by tangential flow filtration (TFF) and ultracentrifugation. Briefly, cell debris was removed from CM by centrifugation at 2,500× *g* for 20 min at 18°C (Centrifuge Model 5810 R, Eppendorf, Hamburg, Germany). The supernatant was filtered through a 0.22 μm Stericup filter (Merck) and the clarified CM was reduced to 60 ml by TFF using a 100 kDa molecular mass cut-off column (Spectrum Labs-Repligen, Breda, The Netherlands). Concentrated CM was further centrifuged at 120,000× *g* for 180 min at 18°C in a Sorvall model WX-80 ultracentrifuge using a fixed-angle rotor model Fiberlite F37L-8x100 to pellet hUC-MSC-EVs. The resulting pellets were washed gently once with PBS and subsequently resuspended in Ringer’s Lactate (Fresenius-Kabi) in an appropriate volume to achieve a dose of 20–40 × 10^6^ cells secretome equivalent/ml. The resulting EV suspension was clarified by centrifugation at 3,000× *g* for 10 min at 4°C (Centrifuge Model 5810 R, Eppendorf) and sterile filtered through a 0.22 μm Stericup filter (Merck). Dilution was made from 2 × 10^12^ particle/ml stock and aliquots were stored at −80°C until use.

### Animal Groups

Experiments were performed in conformity with the Directive 2010/63/EU of the European Parliament and of the council of 22 September 2010 on the protection of animals used for scientific purposes and were approved by the Federal Ministry of the Republic of Austria for Education, Science and Research (BMBWF-66.019/0036-V/3b/2018).

Female F344-rats of 10–12 weeks of age (140–190 g body weight) were purchased from Charles River Laboratories (Sulzfeld, Germany) and kept for at least 4 weeks in the animal facility to acclimatize to the handling by the experimenters. Prior to surgery, rats were randomly divided into three treatment groups each comprising 26 rats that would receive acutely after contusion *via* intra-parenchymal (i.pa.) injection either: (a) 2 μl of Ringer-lactate (i.pa. vehicle) or (b) 2 μl of Ringer-lactate containing 1.5 × 10^9^ extracellular vesicles (i.pa. EVs) into the SCI lesion site, or (c) 100 μl of Ringer-lactate solution containing 1.5 × 10^9^ extracellular vesicles intravenously (i.v. EVs) *via* tail vein injection. In addition, a fourth group (sham) was composed of sham-operated rats that only underwent laminectomy (*n* = 26). Every treatment group was additionally divided into three time points of analysis; i.e., 24 h (*n* = 6), 14 days (*n* = 10) and 56 days (*n* = 10). Nine rats were excluded from the analyses due to inadequate contusion, because they died during surgery or due to post-surgery complications requiring euthanasia (Sham: *n* = 1, i.pa. vehicle: *n* = 3; i.pa. EVs: *n* = 1; i.v. EVs: *n* = 4). Experimenters were blinded to the content of injections and treatment groups until the end of the data acquisition and analysis.

### Surgeries

The operative narcosis was obtained by an intra-muscular injection of a cocktail of medetomidine hydrochloride (Narcostart 150 μg/kg body weight), Midazolam (Midazolam Accord 2 mg/kg body weight), and Fentanyl (Fentanyl-Janssen 10 μg/kg body weight). Body temperature was maintained at 37°C *via* a rectal probe-coupled heating pad. O_2_ saturation and pulse were monitored using a pulse-oximeter (Emka Technologies, Paris, France). A dorsal laminectomy was performed at thoracic level 8 (Th8) leaving the exposed underlying dura mater intact. The neighboring vertebrae (Th7 and Th9) were fixed on the foramina intervertebralia using two Adson forceps. Using an impactor (Infinite Horizon, Precision System and Instrumentation PSI, Fairfax Station, VA, USA), a contusion of 200 kdyn was applied on the spinal cord at Th8 level. Force applied and displacement of tissue were recorded, and only rats with contusion force of 200 kdyn and approximately 1,000 μm of displacements were included in the study.

Immediately after contusion, 2 μl of either Ringer-lactate or Ringer-lactate containing EVs were slowly injected into the spinal cord lesion site at midline with a depth of 0.9 mm using a pulled-glass micropipette mounted on a stereotaxic apparatus (Stoelting, Wood Dale, IL, USA). The third group of rats received an intravenous application of EVs *via* tail vein injection. The rats belonging to the sham group underwent only a laminectomy. Post-operative analgesia was provided directly after surgery and daily for 5 days with meloxicam [1 mg/kg body weight subcutaneous (s.c.)]. On the first two days post-surgery, rats additionally received buprenorphine (0.03 mg/kg body weight s.c.) twice per day. To prevent the occurrence of infection, enrofloxacin (10 mg/kg body weight) was administered s.c. on the day of surgery and daily thereafter, until the 5th day post-surgery. The bladder was manually voided 2–3 times per day. Rats with tSCI were housed on special soft bedding (Arbocell Comfort White bedding, Rettenmaier Austria GmbH, Vienna, Austria). Food and water were freely accessible at a lowered height in the cages.

### Motor and Sensory Tests

At least 2 weeks of training prior to the surgery were used to familiarize the rats with the different tests employed. Additionally, the last training pre-surgery served to establish a baseline for comparison of performance measured after surgery and following treatment.

#### BBB Score

The motor function of hindlimbs was assessed using the non-linear BBB-score scale (Basso et al., 1995), which ranges from (0) total paralysis to (21) normal locomotion. Accordingly, rats moving freely within a 1-m diameter arena were scored for 4 min by two observers blinded to group belonging. Measurements were performed on days 1, 4, 7, 11, 14 and then once per week until the 8th week post-injury. Scores of left and right hindlimbs did not differ significantly, and their means have been used for each time point. BBB sub-scores (ranging from 0 to 13) were also calculated to quantify recovery based on toe clearance, paw position, trunk stability and tail use, independently of forelimb–hindlimb coordination (Popovich et al., 2012).

#### Horizontal Ladder Walk Test

Video recordings were acquired for each rat as they traveled across a horizontal ladder (1 m length, 3 mm rung diameter, 5–20 mm rung irregular spacing) from a neutral cage to reach their home cage with littermates. Total left and right hindlimb correct steps and foot-faults (missteps) were assessed by one observer. The test was performed prior to the surgery, to establish a baseline score, and at 4 and 8 weeks after tSCI. For each time point, the rats walked three times across a ladder with different rung patterns for each repetition. The percentage of missteps was calculated for each rat and averages were used to compare groups.

#### CatWalk

Gait analyses of voluntary locomotion were performed using the CatWalk XT system (Noldus Information Technology, Wageningen, Netherlands). Every session consisted of six valid runs defined as transit across the recording window without pause. Sessions were performed two times prior to surgery and thereafter at weeks 3, 5, and 7. Each paw was documented individually. In addition to the evaluation of single gait parameters, we compared the treatment groups using a p(LDA) we recently described (Timotius et al., 2021), which is a linear discriminant analysis providing a weighted combination of the nine most SCI-affected Catwalk parameters. Importantly, two rats of the vehicle group had to be excluded from the analysis for the 3 weeks post-injury time point since their level of locomotor recovery, leading to insufficient stepping ability, was too low to be analyzed using the CatWalk system.

#### Plantar Test Hargreave’s Method

Rats were first placed in the recording chambers for 20 min of acclimation (Plantar test device from Ugo Basile, Gemonio, Italy). An infrared source (65 Watt) was focused on the plantar surface of the hind paws and the “time to withdrawal” from the heat stimulus was recorded. A cut-off at 15 s was set to avoid burn injury. The plantar test was performed prior to the surgery, as the baseline, and then at 4, 6, and 8 weeks post-injury. Four measurements for each hind paw were recorded at each time point. Scores of left and right hind paws did not differ significantly, and their means have been used for each time point.

### Bladder Function Assessment

Following tSCI, the urinary bladder of rats needed to be emptied manually up to three times per day until a reflex voiding appeared. This progressive functional recovery was monitored during the 8 weeks of recovery based on the average volume of urine that needed to be manually voided during the daily care. The “bladder functionality score” was defined using the bladder size assessed by palpation: Large 1 point, Medium/Large 2 points, Medium 3 points, Small/Medium 4 points, Small 5 points, Very Small 6 points, and Empty 7 points. A daily mean score was calculated for each rat.

### Histology

On day 14 or day 56 after injury (14 dpi or 56 dpi), rats were deeply anesthetized by intraperitoneal injection of ketamine (273 mg/kg body weight), xylazine (7.1 mg/kg body weight), and acepromazine (0.625 mg/kg body weight) and transcardially perfused with 0.9% NaCl containing 10 unit/ml heparin (Sigma), followed by 0.1 M phosphate-buffered 4% paraformaldehyde, pH 7.4. Following perfusion, spinal cords were dissected and further post-fixed for 1 h at room temperature in the same paraformaldehyde solution. Tissues were then washed three times and stored in PBS. Prior to histological analysis, four spinal cords from each experimental group were randomly selected and processed for MRI and μCT imaging.

### Magnetic Resonance Imaging (MRI)

Magnetic resonance imaging was performed on a Bruker BioSpec 7 T system (BioSpec 70/20 USR with Paravision 6.0.1. software version, Bruker BioSpin, Ettlingen Germany) in a Tx/Rx configuration, using an 86 mm transmit volume coil (MT0381, Bruker Biospin, Germany) for transmitting (Tx) and a 2-element mouse brain surface coil (MT0042, Bruker Biospin, Germany) for receiving (Rx). Spinal cords (*n* = 4 for each group) were placed in a standard 15 ml Falcon tube and held close to the coil with a custom-made holder. To remove the background signal, scanned samples were immersed in Fomblin (Solvay, Brussels, Belgium).

The scan protocol consisted of high-resolution anatomical scans (FOV 24.0/4.0/5.2 mm, 80 μm isotropic voxels, producing an image size of 300 × 50 × 60 pixels) and a diffusion tensor measurement scan (FOV 24.0/4.2/5.1 mm, 150 μm isotropic voxels, image size 240 × 28 × 34 pixels). Sagittal slice orientation, corresponding to the smallest sample dimension, was selected in order to minimize scan time. Two anatomical scans were acquired for volumetric analysis: T2-weighted (T2), and T1-weighted with Inversion Recovery (T1-IR). A diffusion-tensor scan (DTI) was performed with a DTI-EPI sequence. Optimal field homogeneity for the DTI-EPI scan was achieved by additional localized shimming using MAPSHIM algorithm.

The timing of the inversion recovery pulse in T1-IR sequence was selected based on prior pilot scans. The optimal timing partially suppresses gray and white matter and completely attenuates scar tissue, while preserving signal from liquids, thus enabling to individuate cysts within the injury site. Cysts were segmented automatically with an in-house developed macro and subsequently corrected by an experienced evaluator. Damaged tissue was delineated manually on consecutive sections based on signal alterations detected on either T2 or T1-IR images. The remaining (non-lesioned) tissue was determined automatically with a simple macro routine, which subtracted the “damaged” ROIs from the whole non-zero signal in every slice. The volumetric analyses included tissue located between 5 mm rostral and caudal from the lesion epicenter. All segmentation was performed in FIJI/ImageJ v.1.53t (Schindelin et al., 2012). One spinal cord from the i.pa. EVs group was excluded from *ex vivo* volumetric analysis due to distortion in the acquisition.

Diffusion tensor data was reconstructed using diffusion tensor imaging (DTI) algorithm in DSI Studio^1^. Prior to reconstruction, images were inspected for distortions and imaging artifacts, after which one sample from the i.pa. EV-treated group was exempt from further analysis. Where needed, eddy current and motion artifact correction were applied using DSI Studio built-in modules. The B-table was checked by an automatic quality control routine to ensure its accuracy (Schilling et al., 2019). The tracking index for generating tracts was fractional anisotropy (FA), and seeds with any orientation and position within the seed voxel were taken as starting points of tracts. The tracking threshold was set to 0.35, differential tracking threshold 0.1, angular threshold 30°, step size 0.06 mm, and minimal fiber length 1 mm.

The number of tracts was measured along the spinal cord samples over a range of equidistant regions of interest placed every 0.75 mm and extending 4.50 mm on either side of the impact epicenter. The epicenter was determined from a high-resolution T2 image as the thinnest portion of the sample in the sagittal plane. For every region, the number of tracts was measured twice, interchanging the “Seed” and “ROI” attributes (i.e., tract start/tract end) in the reconstruction algorithm. The final number of tracts between the two ROIs was calculated as the arithmetic mean and assigned to the midpoint between the two ROIs. To minimize the effect of distortions due to magnetic field inhomogeneity (air bubbles or poor shimming) on measurement results, the number of tracts counted between neighboring points were compared to that measured between second neighbor points. Regions displaying strong deviation between two measurements were excluded from the analysis.

### Micro-Computed Tomography (μCT)

#### μCT Imaging

Spinal cords (*n* = 4 for each group) were contrasted prior to scanning with incubation in Accupaque-350 (GE Healthcare, Munich, Germany) diluted 1:2 in PBS for 48 h. The spinal cords were then placed on 2 mm thick and 4 mm wide extruded polystyrene boards (XPS) and fixed using parafilm. The mounted spinal cords were placed upright and evenly spaced around the inner wall of a cylindrical sample holder with 19 mm diameter and held in place with pieces of XPS boards. Up to four spinal cord samples were scanned at the same time. Scans were performed at 70 kVp with 85 μA and a 0.5 mm Al Filter. A total of 3,400 projections/180° were integrated 4 times for 650 ms and averaged. The scans were reconstructed to an isotropic resolution of 6 μm. Approximately 21 mm of spinal length was scanned with the lesion epicenter positioned in the center of the scan.

The scans were converted to DICOM slices and evaluated using Fiji (ImageJ v1.53a; Schindelin et al., 2012). The spinal cords were rotated and aligned on the Z-Axis of the image using the rotate function of TransformJ (version 2016/01/09). The images were cropped to the XY extent of the spinal cord and in the Z direction to at least 5 mm above and below the lesion epicenter. Due to damage during tissue manipulation, a rat of the sham group was excluded.

#### Classification of Spinal Tissue

The outer borders of the spinal cord and borders of the damaged tissue were drawn using the lasso and polygon tools in ImageJ. These selections were interpolated along the Z-Axis using the Interpolate Regions of Interest (ROIs) function of the ROI Manager. The interpolated ROI was then manually validated slice by slice. Classification of damaged tissue was validated and corrected in the orthogonal views if necessary.

#### Classification of Cyst

Inside the damaged tissue ROI, cysts were defined with a threshold adjusted for each image, based on the intensity of the staining solution. Each scan was filtered using a 3D Gauss filter with a sigma of 4 (24 μm) followed by 3D unsharp masking (sigma 4, strength 0.6). The thresholding resulted in a binary mask which was converted to selections and added to the ROI manager.

#### Volumetry

The classifications of the spinal cord, damaged tissue, and cyst were combined into a single grayscale classification image with different intensity levels for background, the spinal tissue, damaged tissue, and cyst. Measurements were performed on this classification image and one transversal segment was measured in 10 slice segments (total 60 μm). The average cross-section area of the spinal cord, damaged tissue, and cyst was measured for each segment. For comparison between samples, the spinal cords were aligned according to the lesion epicenter.

### Immunohistology

A segment of 15 mm centered on the lesion was selected and transferred into 0.1 M phosphate-buffered 30% sucrose solution for 72 h (*n* = 5–6 per group and time point). Then, samples were embedded in OCT embedding compound (Tissue-Tek, Sakura, Umkirch, Germany) and frozen in 2-methylbutane over liquid nitrogen. Using a cryostat (Leica CM1950), coronal sections of 15 μm were collected in 10 series (each containing every 10th section) on Superfrost Plus microscope slides (Thermo Scientific, Vienna, Austria).

For immunohistological analyses, sections were washed with PBS + 0.1% Tween-20 (Sigma-Aldrich). The blocking solution was composed of PBS containing 1% bovine serum albumin (Sigma-Aldrich), 0.2% fish skin gelatin (Sigma-Aldrich), and 0.1% Tween-20. The primary antibodies: guinea-pig anti-GFAP (1:500; Progen, Heidelberg, Germany), goat anti-Iba1 (1:300; Abcam, Cambridge, UK), rabbit anti-collagen I (1:100; Abcam), goat anti-ChAT (1:100; Novus Biologicals, Abingdon, UK), and rabbit anti-NG2 chondroitin sulfate proteoglycan (1:200; Merck/Millipore) were diluted in blocking solution and applied overnight at 4°C. The secondary antibodies: Alexa Fluor 568 donkey anti-rabbit (1:1,000; Invitrogen, Vienna, Austria), Alexa Fluor 647 donkey anti-guinea pig (1:1,000; Dianova, Hamburg, Germany), and Alexa Fluor 568 donkey anti-goat (1:1,000, Molecular Probes, Vienna, Austria) were applied overnight at 4°C. Nuclei were stained using 4’6-diamidino-2-phenylindole (DAPI; 0.5 μg/ml, Sigma-Aldrich). Finally, sections were mounted with fluorescent mounting medium (ProLong Gold, Thermo Fisher Scientific) and were examined using a confocal fluorescence microscope (Zeiss LSM710) or a slide scanner (Olympus VS120).

### Histological Analyses

#### Spared Tissue

Immunodetection GFAP was used to distinguish intact neural tissue from the necrotic and scarred tissue, and therewith calculated the volume of remaining spared spinal cord tissue, taking the sham rats as reference. The volume of spinal spared tissue was calculated within a segment ranging from 3,150 μm caudal to 3,150 μm rostral of the lesion epicenter based on one section every 1,050 μm. Using the Fiji ImageJ software (Schindelin et al., 2012), the area of intact neural tissue was manually delineated on the micrographs. The total spared volume corresponded to the sum of the volumes extrapolated from the intact areas measured on each section and the distance between the sections (1,050 μm).

#### Microglia/Macrophages Cell Density

The intensity of the inflammatory response was determined based on the density of Iba1-expressing cells in the gray matter of the ventral horn (volume of interest: 425 μm × 425 μm × 15 μm), by counting all Iba1^+^ cells with a nucleus located in the volume of interest. The densities were calculated on the sections located at 2,100 μm and 3,150 μm, both rostral and caudal from the injury epicenter, and pooled together (four sections in total per rat). The tissue destruction at positions closer to the epicenter did not allow reliable quantification in the ventral horn. Additionally, corresponding positions along the rostral-caudal axis of the spinal cord of sham group rats were analyzed.

### Reactive Gliosis and Scarring

GFAP, NG2, and collagen I expression were quantified according to the area stained by immunodetection on the tissue sections. Immunofluorescence images were acquired with fixed parameters and binarized as previously described (Romanelli et al., 2019). For the analysis of reactive gliosis, the quantification of GFAP expression was performed in one ventral horn (area of interest: 425 μm × 425 μm) on sections located at 2,100 μm and 3,150 μm, both rostral and caudal from the injury epicenter, and pooled together (four sections in total per rat). The ventral horn could not be reliably defined in regions closer to the epicenter, which was therefore not included in the analysis. Scarring was investigated based on the deposition of NG2 chondroitin sulfate proteoglycan and collagen I and quantified according to the area stained by immunodetection on the tissue sections positioned at the epicenter as well as 1,050 μm, 2,100 μm, and 3,150 μm rostral and caudal from the epicenter (seven sections in total per rat). Additionally, corresponding positions of sham group rats were analyzed along the rostral-caudal axis of the spinal cord. The area covered by the staining was determined using the Fiji ImageJ software (Schindelin et al., 2012).

#### Number of Choline Acetyltransferase-Expressing Motor Neurons

The number of cells expressing choline acetyltransferase (ChAT) was determined on sections located 2,100 μm, and 3,150 μm, both rostral and caudal from the injury epicenter of each rat (four sections per rat). The number of ChAT^+^ cells was counted in both ventral horns and results were pooled together. Additionally, corresponding positions along the rostral-caudal axis of the spinal cord of sham group rats were analyzed.

### Cytokine Expression in the Spinal Cord and Serum After tSCI

One day after injury or laminectomy, rats (*n* = 6 per group) were deeply anesthetized by intraperitoneal injection of ketamine (273 mg/kg body weight), xylazine (7.1 mg/kg body weight), and acepromazine (0.625 mg/kg body weight), and a maximal volume of cardiac blood was collected. Following decapitation, the spinal cords were rapidly dissected for RNA extraction.

Total RNA of each spinal cord was isolated from a 1 cm segment centered on the lesion site, or the corresponding position in the sham group, using the TRIzol reagent (Sigma-Aldrich) according to the manufacturer’s protocol. RNA concentrations were determined with a NanoVue plus (GE Healthcare, UK). RNAs were reverse transcribed into first-strand cDNA using the iScript TM Reverse Transcription Supermix for RT-qPCR (Bio-Rad Laboratories, Vienna, Austria) according to the manufacturer’s protocol. Quantitative gene expression analyses were performed using TaqMan RT-PCR technology. Technical duplicates containing 10 ng of reverse transcribed RNA were amplified with the GoTAQ Probe qPCR Master Mix (Promega) using a two-step cycling protocol (95°C for 15 s, 60°C for 60 s; 50 cycles, Bio-Rad CFX 96 Cycler). The following validated exon-spanning gene expression assays were employed: pPIA Rn.PT.39a.22214830 and TBP Rn.PT.39a.22214837 from Integrated DNA Technologies (IDT, Leuven, Belgium); arginase 1 Rn00691090; IL-6 Rn01410330; IL-18 Rn01422083; caspase 1 Rn00562724; TNF-α Rn99999017; NLRP1a Rn01467482 and NLRP3 Rn04244620 from ThermoFisher; and IL-1β NM_031512.2 from Sino Biological (Eschborn, Germany).

The relative expression levels of the target genes were normalized on two validated reference genes, Peptidylprolyl isomerase A (PPIA) and TATA-binding protein (TBP). C_q_ values were analyzed using QBasePlus v. 3.2 (Biogazelle NV, Zwijnaarde, Belgium). Expression of target genes in control and treatment conditions were normalized to represent the relative expression in terms of “fold changes”.

Following cardiac blood collection, the serum was isolated by centrifugation and stored at −80°C until analyses. The MSD V-Plex Proinflammatory Panel 2 Rat Kit (Rockville, Maryland, USA) was used to measure serum concentrations of IL-1β, IL-4, IL-6, IL-10, IL-13, TNF-α, IFN-γ, and KC/GRO according to the manufacturer’s instructions. Samples were run in duplicates. Plates were quantified with a MESO QuickPlex SQ 120 (Meso Scale Discovery), and data were analyzed using DISCOVERY WORKBENCH Data Analysis software (Meso Scale Discovery).

### Statistics

Statistical analyses were performed using the Prism 8 software (GraphPad, San Diego, CA, USA). One-way ANOVA and two-way ANOVA tests were performed, followed by a Tukey *post hoc* test. Repeated measures one-way ANOVA or two-way ANOVA was used where indicated. Baseline scores pre-tSCI were not included in group analyses of treatment effect over time. The measurements performed on the three SCI-groups were compared using one-way ANOVA, followed by Tukey *post hoc* test, to detect the effects of EV treatments following SCI (significances marked with asterisks on the graphs). Moreover, when data were also acquired for the sham group, the SCI and sham groups were compared with an additional one-way ANOVA, followed by Tukey *post hoc* test, to detect effect related to SCI (significances marked with pound signs on the graphs). Statistical significance was assumed for *p* < 0.05. Data are shown as mean ± standard deviation.

## Results

Using a moderate to severe contusion tSCI rat model (200 kdyn, Infinite Horizon Impactor) previously described in Romanelli et al. (2019), we compared the effects of EVs applied intravenously (i.v.) or injected intra-parenchymal (i.pa.) directly in the lesion site. The intra-parenchymal application allows for the rapid delivery of EVs at the lesion in high concentrations. Here, we analyzed the impact of EV application immediately after tSCI and in particular the impact of these two administration routes on the functional and structural outcomes.

### Acute Intralesional Application of EVs Improves Functional Outcomes After tSCI

**Figure 1:**
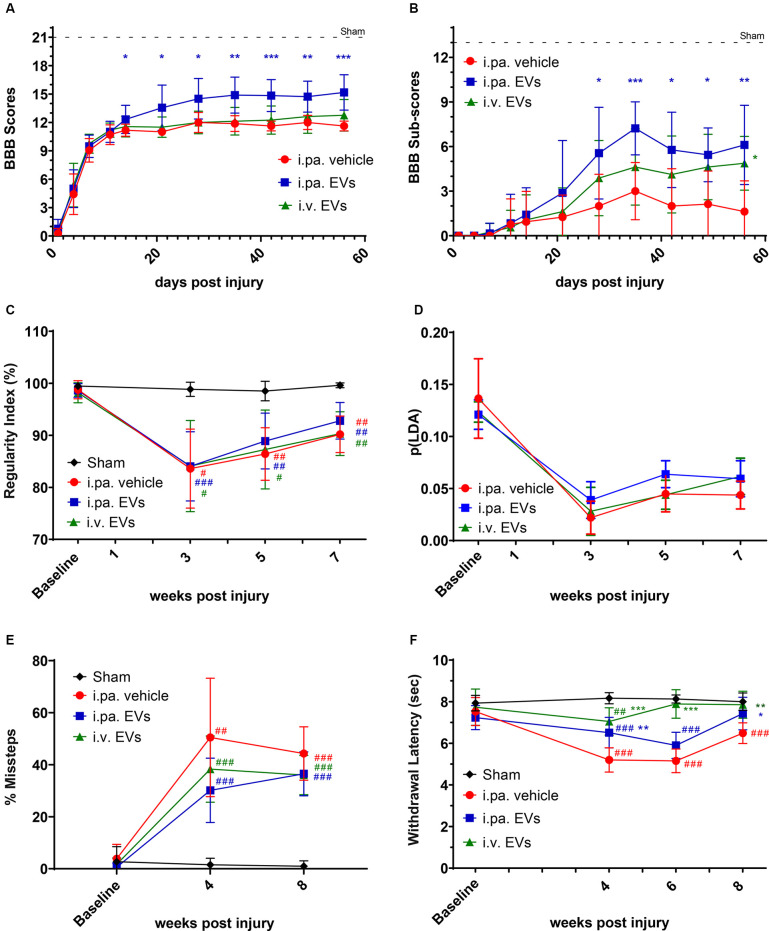
Acute intralesional application of EVs improved functional recovery after tSCI. The locomotor function of rats was monitored by **(A)** BBB scores and **(B)** sub-score in the tSCI groups. **(C)** Inter-limb coordination was assessed by Catwalk regularity index. **(D)** The p(LDA) was calculated to combine the nine most SCI-affected Catwalk parameters. **(E)** Skilled walking was tested with the horizontal ladder walk test and the percentage of step cycles containing missteps was monitored over time. **(F)** Thermal sensitivity was assessed according to the withdrawal latencies to a thermal stimulus using the plantar test Hargreaves’ method. Statistical differences to sham group using two-way ANOVA and Tukey *post hoc* test: ^(#)^*p* < 0.05, ^(##)^*p* < 0.01, ^(###)^*p* < 0.001, statistical differences to i.pa vehicle group using two-way ANOVA and Tukey *post hoc* test on tSCI groups (without sham): ^(*)^*p* < 0.05, ^(**)^*p* < 0.01, ^(***)^*p* < 0.001. EVs, extracellular vesicles; tSCI, traumatic spinal cord injury; BBB, Basso-Beattie-Bresnahan.

Recovery of locomotor function after tSCI was monitored according to the widely used locomotor Basso-Beattie-Bresnahan (BBB) scale (maximal score 21; Basso et al., 1995). On the first day post-tSCI, hindlimbs of rats were nearly paralyzed (BBB score 0.5 ± 0.7; Figure 1A). Over the following days, the mobility of joints and the walking pattern progressively recovered in all tSCI groups (Figure 1A ). Strikingly, from the 2nd week post-injury onward, the recovery of locomotion in i.pa. EV-treated rats was significantly more robust compared to the recovery following vehicle application. At 56 days post-injury, the BBB score reached 15.2 ± 1.9 in i.pa. EV-treated rats, which corresponds to a consistent forelimb–hindlimb coordination with predominant parallel paw placement at the initial floor contact (Figure 1A; Table 1). In contrast, the BBB scores of vehicle and i.v. EV-treated rats only reached 11.6 ± 0.5 and 12.7 ± 1.7 respectively, which corresponds to consistent weight-supported plantar steps with occasional forelimb–hindlimb coordination (Figure 1A; Table 1).

**Table 1:** Summary of functional tests.

	**Sham**	**i.pa. vehicle**	**i.pa. EVs**	**i.v. EVs**
**BBB scores**			
1 day post-injury		0.3 ± 0.5 *n* = 17	0.8 ± 1.0 *n* = 19 n.s.	0.3 ± 0.5 *n* = 16 n.s.
4 days post-injury		4.4 ± 2.1 *n* = 17	5.0 ± 2.0 *n* = 19 n.s.	5.4 ± 2.3 *n* = 16 n.s.
7 days post-injury		9.1 ± 1.2 *n* = 17	9.5 ± 1.2 *n* = 19 n.s.	9.7 ± 1.0 *n* = 16 n.s.
11 days post-injury		10.7 ± 1.0 *n* = 17	11.0 ± 1.1 *n* = 19 n.s.	11.2 ± 0.7 *n* = 16 n.s.
14 days post-injury		11.2 ± 0.6 *n* = 17	12.3 ± 1.5 *n* = 19*	11.6 ± 1.1 *n* = 16 n.s.
21 days post-injury		11.0 ± 0.0 *n* = 8	13.6 ± 2.4 *n* = 9*	11.5 ± 1.1 *n* = 8 n.s.
28 days post-injury		12.0 ± 1.1 *n* = 8	14.5 ± 2.2 *n* = 9*	12.0 ± 1.2 *n* = 8 n.s.
35 days post-injury		11.9 ± 0.8 *n* = 8	14.9 ± 1.9 *n* = 9**	12.1 ± 1.5 *n* = 8 n.s.
42 days post-injury		11.6 ± 0.5 *n* = 8	14.8 ± 1.7 *n* = 9***	12.2 ± 1.5 *n* = 8 n.s.
49 days post-injury		12.0 ± 0.8 *n* = 8	14.7 ± 1.6 *n* = 9**	12.6 ± 1.8 *n* = 8 n.s.
56 days post-injury		11.6 ± 0.5 *n* = 8	15.2 ± 1.9 *n* = 9***	12.7 ± 1.7 *n* = 8 n.s.
**BBB sub-scores**			
1 day post-injury		0.0 ± 0.0 *n* = 17	0.0 ± 0.0 *n* = 19 n.s.	0.0 ± 0.0 *n* = 16 n.s.
4 days post-injury		0.0 ± 0.0 *n* = 17	0.0 ± 0.0 *n* = 19 n.s.	0.0 ± 0.0 *n* = 16 n.s.
7 days post-injury		0.0 ± 0.0 *n* = 17	0.1 ± 0.7 *n* = 19 n.s.	0.2 ± 0.7 *n* = 16 n.s.
11 days post-injury		0.8 ± 1.7 *n* = 17	0.8 ± 1.9 *n* = 19 n.s.	0.6 ± 1.1 *n* = 16 n.s.
14 days post-injury		0.9 ± 2.0 *n* = 17	1.4 ± 1.8 *n* = 19 n.s.	1.1 ± 1.7 *n* = 16 n.s.
21 days post-injury		1.2 ± 1.5 *n* = 8	2.9 ± 3.5 *n* = 9 n.s.	1.6 ± 1.6 *n* = 8 n.s.
28 days post-injury		2.0 ± 2.1 *n* = 8	5.6 ± 3.1 *n* = 9*	3.9 ± 2.5 *n* = 8 n.s.
35 days post-injury		3.0 ± 1.9 *n* = 8	7.2 ± 1.8 *n* = 9***	4.6 ± 2.6 *n* = 8 n.s.
42 days post-injury		2.0 ± 2.5 *n* = 8	5.8 ± 2.5 *n* = 9*	4.1 ± 2.6 *n* = 8 n.s.
49 days post-injury		2.1 ± 2.2 *n* = 8	5.4 ± 1.8 *n* = 9*	4.6 ± 2.2 *n* = 8 n.s.
56 days post-injury		1.6 ± 2.1 *n* = 8	6.1 ± 2.7 *n* = 9**	4.9 ± 1.8 *n* = 8*
**% Regularity index (CatWalk) **			
Baseline	99.5 ± 0.6 *n* = 9	99.1 ± 1.6 *n* = 8 n.s.	98.6 ± 1.3 *n* = 9 n.s., n.s.	98.4 ± 1.9 *n* = 8 n.s., n.s.
21 days post-injury	98.9 ± 1.4 *n* = 9	83.6 ± 7.6 *n* = 6^#^	84.0 ± 6.6 *n* = 9 n.s.,^###^	84.6 ± 8.3 *n* = 8 n.s.,^##^
35 days post-injury	98.5 ± 1.9 *n* = 9	77.8 ± 16.8 *n* = 8^#^	88.9 ± 5.4 *n* = 9 n.s.,^##^	86.6 ± 7.3 *n* = 8 n.s.,^##^
49 days post-injury	99.6 ± 0.5 *n* = 9	86.3 ± 8.5 *n* = 8^#^	92.8 ± 3.5 *n* = 9 n.s.,^##^	89.5 ± 4.6 *n* = 8 n.s.,^##^
**p(LDA) (Catwalk)**			
Baseline		0.135 ± 0.041 *n* = 8	0.121 ± 0.014 *n* = 9	0.124 ± 0.010 *n* = 8
21 days post-injury		0.027 ± 0.013 *n* = 6	0.039 ± 0.018 *n* = 9	0.028 ± 0.023 *n* = 8
35 days post-injury		0.045 ± 0.018 *n* = 8	0.064 ± 0.013 *n* = 9	0.044 ± 0.014 *n* = 8
49 days post-injury		0.045 ± 0.012 *n* = 8	0.059 ± 0.017 *n* = 9	0.062 ± 0.018 *n* = 8
**Missteps (Ladder Walk)**
Baseline	2.7 ± 5.8 *n* = 10	3.8 ± 5.5 *n* = 8 n.s.	0.6 ± 1.7 *n* = 9 n.s., n.s.	1.7 ± 2.4 *n* = 8 n.s., n.s.
28 days post-injury	1.5 ± 2.5 *n* = 10	50.5 ± 22.8 *n* = 8^##^	30.1 ± 12.4 *n* = 9 n.s.,^###^	38.3 ± 12.7 *n* = 8 n.s.,^###^
56 days post-injury	1.0 ± 2.1 *n* = 10	44.3 ± 10.3 *n* = 8^###^	36.5 ± 8.4 *n* = 9 n.s.,^###^	36.0 ± 7.5 *n* = 8 n.s.,^###^
**Withdraw Lat. (s) (Hargreaves Test)**			
Baseline	7.9 ± 0.4 *n* = 10	7.5 ± 0.7 *n* = 8 n.s.	7.2 ± 0.6 *n* = 9 n.s.,^#^	7.7 ± 0.9 *n* = 8 n.s., n.s.
28 days post-injury	8.2 ± 0.3 *n* = 10	5.2 ± 0.6 *n* = 8^###^	6.5 ± 0.7 *n* = 9^**,###^	7.1 ± 0.7 *n* = 8 ^***,##^
42 days post-injury	8.1 ± 0.2 *n* = 10	5.1 ± 0.6 *n* = 8^###^	5.9 ± 0.6 *n* = 9 n.s.,^###^	7.9 ± 0.7 *n* = 8 ***, n.s.
56 days post-injury	8.0 ± 0.4 *n* = 10	6.5 ± 0.5 *n* = 8^###^	7.4 ± 0.8 *n* = 9*, n.s.	7.9 ± 0.6 *n*=8**, n.s.

Summary of measurements addressing motor and sensory recovery. The results are displayed as means ± standard deviations. Statistically significant differences to the sham group are marked with ^(#)^*p* < 0.05, ^(##)^*p* < 0.01, and ^(###)^*p* < 0.001. Statistically significant differences to the vehicle are marked with ^(*)^*p* < 0.05, ^(**)^*p* < 0.01, and ^(***)^*p* < 0.001. Non-significant differences are marked with n.s..

Furthermore, we analyzed the BBB sub-score, which combines various parameters of locomotion, independently of forelimb–hindlimb coordination (maximal score 13). Starting at 28 days post-injury, the BBB sub-score was significantly higher in rats that received i.pa. EV application, as compared to the vehicle-treated rats (Figure 1B; Table 1). Importantly, at 56 days post-injury, the BBB sub-scores of i.pa. and i.v. EV-treated rats (6.1 ± 2.7 and 4.9 ± 1.8, respectively) were significantly higher than the sub-score of vehicle-treated rats (1.6 ± 2.1, *p* < 0.001), and no significant difference was detected between the EV-treated groups at this time point.

The CatWalk XT system was further used to analyze limb coordination during voluntary locomotion. Assessment of the step cycle regularity after tSCI confirmed the strongly impaired coordination 3 weeks after injury (Figure 1C; Table 1). As expected, sham rats showed a regularity index consistently close to 100%. In the weeks following tSCI, a progressive, but incomplete recovery of interlimb coordination, as well as a decrease of the inter-individual variability, were observed in all injured groups (Figure 1C; Table 1). Application of EVs, either i.pa. or i.v., did not significantly accelerate the recovery of step cycle regularity, as compared to the vehicle treatment (Figure 1C). At 7 weeks post-injury, the three tSCI groups still presented a regularity index significantly lower than the sham group. In addition, we calculated a p(LDA) representing a weighted combination of the nine CatWalk parameters most affected by contusion SCI (Timotius et al., 2021). As expected, the p(LDA) value decreased significantly after contusion and progressively recovered over time for all treatment groups. Furthermore, 5 weeks after injury, the p(LDA) of i.pa. EVs treated rats was greater in comparison to the vehicle-treated rats, although the difference did not reach significance (i.pa.vehicle: 0.045 ± 0.018; i.pa. EVs: 0.064 ± 0.013; *p* = 0.06; Figure 1D; Table 1).

In addition, we assessed skilled walking with the horizontal ladder walk test. The baseline performance of rats was good and we observed less than 3% of step cycles containing missteps (Figure 1E; Table 1). In contrast, 4 and 8 weeks after tSCI, the percentage of step cycles containing missteps was significantly larger in all three injured groups, as compared to the sham group (Figure 1E; Table 1). After 8 weeks ofs tSCI, the rats treated with EVs performed slightly better than vehicle-treated rats. However, this difference was not statistically significant.

The disturbance of sensory function following tSCI was examined using the plantar test Hargreaves’ method. The shorter hind paws’ withdrawal latencies to thermal stimuli measured 4 weeks following tSCI revealed the emergence of thermal hypersensitivity in all lesioned groups (Figure 1F; Table 1). The withdrawal latencies of both groups treated with EVs normalized during the 8 weeks of recovery and were eventually comparable to the latency measured in the sham group (Figure 1F; Table 1). In contrast, 8 weeks after tSCI, rats that had received the vehicle treatment still withdrew their hind paws significantly faster upon thermal stimulus as compared to the sham group (*p* < 0.001). It is worth noting that 6 weeks post-injury, the rats treated with an i.v. EV application already reacted with withdrawal latencies comparable to the sham group, whereas the latencies measured in the i.pa. EV and vehicle-treated groups were still significantly shorter (*p* < 0.001; Figure 1F; Table 1).

Finally, in addition to the paralysis and partial functional recovery of hindlimb muscles, urinary bladder dysfunction is also observed in this rat model of tSCI. Loss of bladder control and functionality is a devastating problem for a large proportion of SCI patients. We assessed, by palpation, the recovery of bladder voiding capacity over time, based on a scoring system describing the accumulation of urine in the disabled bladder (score ranged from 1 point: very large bladder, to 7 points: empty bladder). The reappearance of a voiding capacity through compensatory reflexes was detected from day 5 after tSCI onward (Supplementary Figure 1). From the 3rd week post-injury until the end of the observation period, the bladder volumes palpated in all groups of injured rats were similar to those of sham rats with intact bladder functionality.

### Acute Intralesional Application of EVs After tSCI Quenches the Inflammatory Response

**Figure 2:**
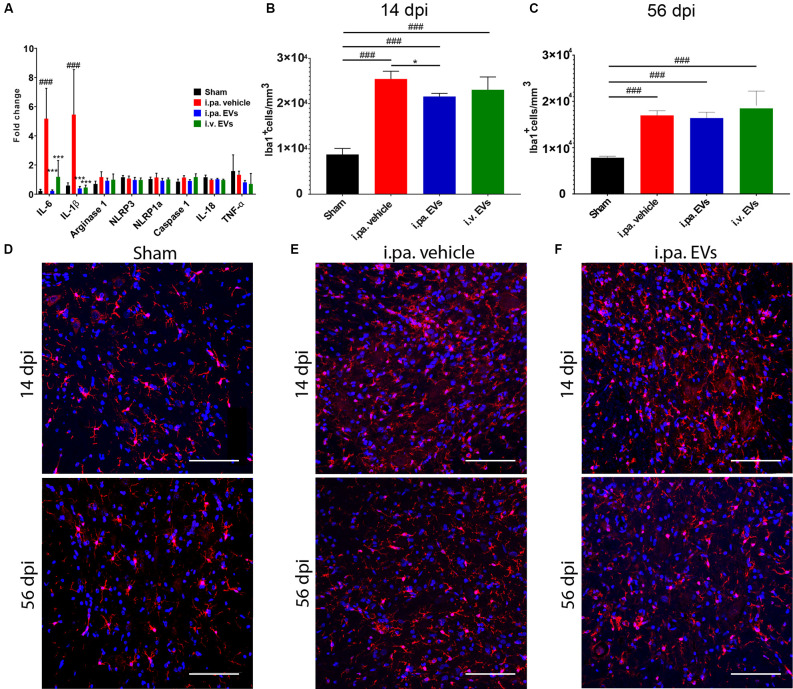
Acute intralesional application of EVs quenched the inflammatory response after tSCI. **(A)** Impact of EV treatment on the expression level of pro-inflammatory genes at the lesion site after tSCI. The density of Iba1-expressing cells was quantified at 14 days **(B)** and 56 days **(C)** after tSCI in the ventral horn. Representative immunodetection of Iba1 in the spinal cord of rats from **(D)** the sham group, **(E)** from the i.pa. vehicle-treated tSCI group and **(F)** from the i.pa. EVs- treated SCI-group (2 mm rostral from the epicenter). Immunodetection of Iba1-expressing microglia (red) was performed 14 days and 56 days post-tSCI. Nuclear counterstain was obtained with DAPI (blue). Scale bar = 50 μm. Statistical differences to sham group using one-way ANOVA and Tukey *post hoc* test: ^(###)^*p* < 0.001, statistical differences to i.pa. vehicle group using one-way ANOVA and Tukey *post hoc* test on tSCI groups (without sham): ^(*)^*p* < 0.05, ^(***)^*p* < 0.001. Days post-injury: dpi.

We examined, 24 h post-injury, the capacity of acute EV applications to install a milder inflammatory environment within the lesioned spinal cord. As we have observed previously (Romanelli et al., 2019), tSCI provokes a strong upregulation of the expression of the pro-inflammatory cytokines IL-1β and IL-6 at the lesion site at this time point. Compared to vehicle-treated rats, the i.pa. application of EVs significantly reduced the induction of IL-1β and IL-6 expression by more than 90% (*p* < 0.001; Figure 2A). A similar reduction in IL-1β expression was also detected following the i.v. application of EVs. However, IL-6 expression was less efficiently suppressed by i.v. application of EVs and showed a reduction of approximately 77% (Figure 2A). In contrast, the expression of arginase 1, TNF-α, NLRP1a, NLRP3, Caspase 1, and IL-18 was neither significantly regulated by tSCI at this time point, nor were their expressions modulated by the application of EVs (Figure 2A).

Furthermore, we investigated the level of circulating inflammatory cytokines in the serum at 24 h post-injury. At this early time point, we could not detect significant differences in the serum concentrations of IL-1β, IL-4, IL-6, IL-10, IL-13, TNF-α, IFN-γ, and KC/GRO between the injured rats treated with vehicle or EVs (data not shown). Hence, at the beginning of the phase of secondary damages, the application of EVs mainly addressed the local inflammatory processes at the lesion site.

To investigate in more detail the impact of EVs on the course of the local inflammatory response, we calculated the density of Iba1-expressing microglia/macrophages in the ventral horns of the lesioned spinal cord during the sub-acute phase (14 days post-injury) and at the beginning of the chronic phase (56 days post-injury; Figures 2B–F; Table 2). Fourteen days after contusion, a significantly higher density of Iba1^+^ cells was detected in the gray matter of the lesioned spinal cord (vehicle group 2.54 × 10^4^ ± 0.17 × 10^4^ Iba1^+^ cells/mm^3^), as compared to the situation observed in sham rats (0.88 × 10^4^ ± 0.13 × 10^4^ Iba1^+^ cells/mm^3^, *p* < 0.001). As shown in Figure 2B, 14 days after tSCI, only the i.pa. EVs significantly dampened (-15%) the accumulation of Iba1^+^ cells (2.16 × 10^4^ ± 0.07 × 10^4^ Iba1^+^ cells/mm^3^, *p* < 0.05), as compared to the vehicle-treated rats. In contrast, no significant difference was observed at this time point between the i.v. EVs (2.31 × 10^4^ ± 0.28 × 10^4^ Iba1^+^ cells/mm^3^) and vehicle-treated rats (Figures 2B,D–F; Table 2). The number of Iba1^+^ cells detected on day 56 post-injury decreased in the vehicle-treated rats by roughly one-third as compared to 14 days post-injury (Figure 2C; Table 2). At this late time point, no treatment-associated differences were observed between the tSCI groups. Nevertheless, all lesioned groups presented significantly higher densities of Iba1-expressing cells than the sham group (*p* < 0.001; Figures 2C–F; Table 2).

**Table 2:** Summary of histological measurements.

	**Sham**	**i.pa. vehicle**	**i.pa. EVs**	**i.v. EVs**
**Iba1^+^ cells/mm^3^**
14 days post-injury	8,780 ± 1323 *n* = 5	25,441 ± 1,712 *n* = 5^###^	21,566 ± 715 *n* = 6^*,###^	23,051 ± 2,837 *n* = 5 n.s.,^###^
56 days post-injury	7,809 ± 333 *n* = 6	16,947 ± 1,028 *n* = 4^###^	16,380 ± 1,272 *n* = 5 n.s.,^###^	18,483 ± 3,110 *n* = 5 n.s.,^###^
**% GFAP+ area**				
14 days post-injury	4.5 ± 0.8 *n* = 5	9.6 ± 1.8 *n* = 5^###^	6.3 ± 1.0 *n* = 6**, n.s.	6.3 ± 0.9 *n* = 5**, n.s.
56 days post-injury	3.8 ± 1.3 *n* = 6	8.6 ± 1.6 *n* = 4^###^	5.7 ± 1.2 *n* = 5*, n.s.	6.3 ± 1.1 *n* = 6^*, #^
**% collagen I area**				
14 days post-injury	0.6 ± 0.1 *n* = 5	9.0 ± 1.8 *n* = 5^###^	6.5 ± 1.0 *n* = 6^**,###^	7.3 ± 0.7 *n* = 5 n.s.,^###^
56 days post-injury	0.6 ± 0.1 *n* = 6	8.5 ± 1.8 *n* = 4^###^	6.4 ± 1.3 *n* = 5 n.s.,^###^	6.4 ± 0.5 *n* = 5 n.s.,^###^
**% CSPG-NG2 area**				
14 days post-injury	3.0 ± 0.8 *n* = 5	10.7 ± 0.9 *n* = 5^###^	8.1 ± 1.3 *n* = 6^**,###^	9.1 ± 0.7 *n* = 5 n.s.,^###^
56 days post-injury	2.4 ± 0.7 *n* = 6	9.5 ± 1.6 *n* = 4^###^	7.7 ± 0.7 *n* = 5 n.s.,^###^	7.6 ± 1.0 *n* = 6 n.s.,^###^
**Spared tissue volume (mm^3^)**				
14 days post-injury	38.3 ± 3.1 *n* = 6	23.5 ± 3.9 *n* = 5^###^	28.1 ± 3.1 *n* = 6 n.s.,^###^	27.9 ± 3.9 *n* = 5 n.s.,^###^
56 days post-injury	41.0 ± 2.9 *n* = 6	17.8 ± 2.4 *n* = 4^###^	22.0 ± 1.9 *n* = 5^*,###^	19.0 ± 2.3 *n* = 6 n.s.,^###^
**Number ChAT+ cells **				
14 days post-injury	60.6 ± 6.4 *n* = 5	47.2 ± 6.7 *n* = 5^#^	47.7 ± 8.5 *n* = 6 n.s.,^#^	36.5 ± 5.4 *n* = 4 n.s.,^###^
56 days post-injury	51.0 ± 3.2 *n* = 6	35.2 ± 12.7 *n* = 4^#^	31.7 ± 6.5 *n* = 4 n.s.,^##^	36.0 ± 8.0 *n* = 6 n.s.,^#^

The results are displayed as means ± standard deviations. Statistically significant differences to the sham group are marked with ^(#)^*p* < 0.05, ^(##)^*p* < 0.01, and ^(###)^*p* < 0.001. Statistically significant to differences to the vehicle are marked with ^(*)^*p* < 0.05 and ^(**)^*p* < 0.01. Non-significant differences are marked with n.s.

### Acute Intralesional Application of EVs Reduces Scarring After tSCI

**Figure 3:**
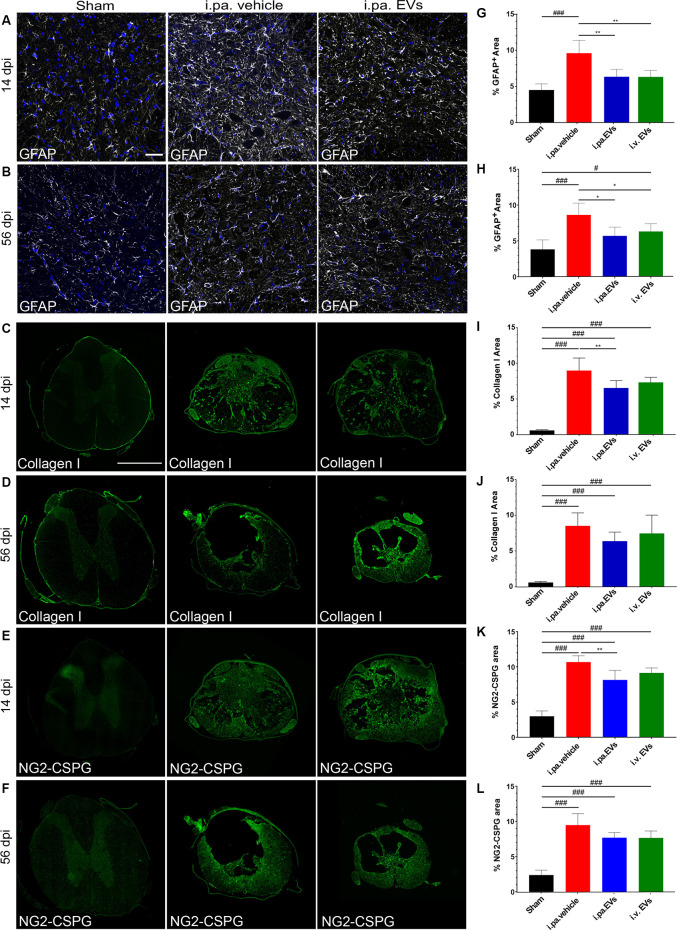
Acute intralesional application of EVs reduced astrogliosis and scarring after tSCI. Immunodetection of GFAP **(A,B)**, collagen I **(C,D)**, and NG2 **(E,F)** in the spinal cord of a sham rat and following tSCI. **(A,B)** Immunodetection of GFAP-expressing astrocytes (white) in the ventral horn of sham rats, i.pa. vehicle-treated tSCI rats and i.pa. EVs-treated tSCI rats at 14 days **(A)** and 56 days **(B)** post-injury (3 mm rostral from lesion epicenter); Nuclear counterstain with DAPI (blue). Scale bar **(A)** = 50 μm. **(C,D)** Immunodetection of collagen I (green) in the spinal cord of sham rats, i.pa. vehicle-treated and i.pa. EVs-treated rats at 14 days **(C)** and 56 days **(D)** post-tSCI at the lesion epicenter. **(E,F)** Immunodetection of NG-2 (green) in the spinal cord of sham rats i.pa. vehicle-treated and i.pa. EVs-treated rats at 14 days **(E)** and 56 days **(F)** post-tSCI at the lesion epicenter. Scale bar **(E)** = 1,000 μm. **(G,H)** Percentage of section area covered by GFAP at 14 days **(G)** and 56 days **(H)** post-tSCI. **(I,J)** Percentage of section area covered by collagen I at 14 days **(I)** and 56 days **(J)** post-tSCI. **(K,L)** Percentage of section area covered NG2 at 14 days **(K)** and 56 days **(L)** post-tSCI. Statistical differences to sham group using one-way ANOVA and Tukey *post hoc* test: ^(#)^*p* < 0.05, ^(###)^*p* < 0.001, statistical differences to i.pa. vehicle group using one-way ANOVA and Tukey *post hoc* test on tSCI groups (without sham): ^(*)^*p* < 0.05, ^(**)^*p* < 0.01. Days post-injury: dpi.

During the sub-acute phase post-tSCI, reactive astrocytes proliferate and build a glial scar at the lesion site that acts as a physical barrier. Furthermore, astrocytes residing in the intact parenchyma proximal to the lesion become hypertrophic and upregulate the expression of the glial fibrillary acidic protein (GFAP; Silver and Miller, 2004; Renault-Mihara et al., 2008; Bradbury and Burnside, 2019). The immunodetection of GFAP can therefore be used to monitor ongoing reactive gliosis (Figures 3A,B,G,H; Table 2). At 14 days post-tSCI, examination in the perilesional area revealed that the percentage of area covered with GFAP had more than doubled in tSCI rats that received the vehicle treatment, as compared to the sham group (*p* < 0.001; Figure 3G; Table 2). In contrast, EV treatment following tSCI significantly lowered the accumulation of GFAP by approximately 35%, compared to the vehicle treatment (*p* < 0.01; Figure 3G; Table 2). The accumulation of GFAP observed at 56 days post-injury was similar to the respective values at 14 days post-injury for all groups. At this late time point, rats that had received EVs after tSCI exhibited significantly less astrogliosis-associated GFAP accumulation compared to vehicle-treated rats (Figure 3H).

Following tSCI, non-cellular components, such as CSPGs (Lemons et al., 1999) and collagen (Tobin et al., 1980), accumulate in the fibroglial scar and create an inhibitory environment for axonal sprouting (Hermanns et al., 2001; Ohtake and Li, 2015). We assessed the impact of early EV applications on the accumulation of collagen I and NG2-CSPGs by immunodetection (Figures 3C–F,I–L). Analysis at 14 days post-injury demonstrated that both administration routes of EVs reduced the deposition of NG2-CSPGs and collagen I compared to rats treated with the vehicle (Figures 3I,K; Table 2). However, only the i.pa. application of EVs reached statistical significance (collagen I *p* < 0.01; NG2 *p* < 0.01). The accumulation of collagen I and NG2-CSPGs detected at 56 days post-injury was comparable to the 14 days time point, but no significant difference was detected between the treatment groups (Figures 3J,L; Table 2).

### Impact of Acute Application of EVs on Structural Integrity After tSCI

**Figure 4:**
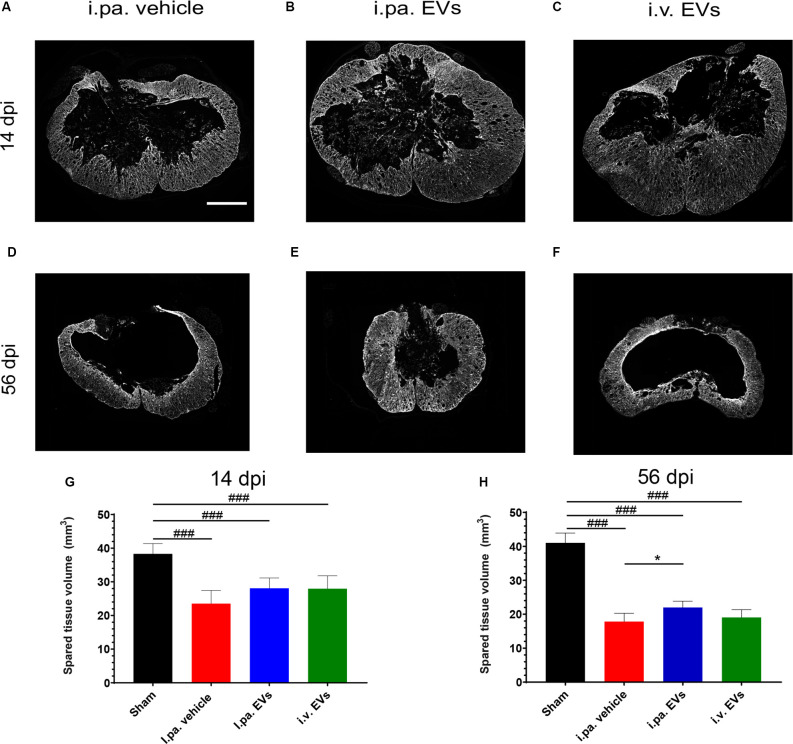
Early intralesional application of EVs spared more parenchyma after tSCI. **(A–F)** Representative micrographs of GFAP immunodetection on spinal cord sections at lesion epicenter at 14 days **(A–C)** and at 56 days post-tSCI **(D–F)**. Scale bar = 500 μm. The total volume of healthy-appearing parenchyma was calculated from 3 mm rostral to 3 mm caudal to the lesion epicenter, or the equivalent position in the sham rats at 14 days **(G)** and 56 days post-tSCI **(H)**. Statistical differences to sham group using one-way ANOVA and Tukey *post hoc* test: ^(###)^*p* < 0.001, statistical differences to i.pa. vehicle group using one-way ANOVA and Tukey *post hoc* test on tSCI groups (without sham): ^(*)^*p* < 0.05. Days post-injury: dpi.

Examination of the spinal cords at 14 days and 56 days post-injury revealed that the lesion epicenter was characterized by a large necrotic cavity surrounded by a thin rim of spared tissue (Figures 4A–F). Taking the sham rats as a reference, we calculated that vehicle-treated rats lost approximately 40% of the spinal tissue within a region comprised of 3,150 μm rostral and 3,150 μm caudal to the lesion epicenter 14 days post-tSCI (Figure 4G; Table 2). At this time point, the volumes of intact parenchyma detected in rats that received EVs were not significantly different from the vehicle-treated rats (Figure 4G; Table 2). In all three tSCI groups, the amount of remaining intact tissue further decreased until 56 days post-injury (Figures 4G,H; Table 2). At this later time point, a significantly larger preservation of tissue was detected in the rats that received i.pa. EV application, compared to the vehicle-treated group (*p* < 0.05; Figure 4H).

**Figure 5:**
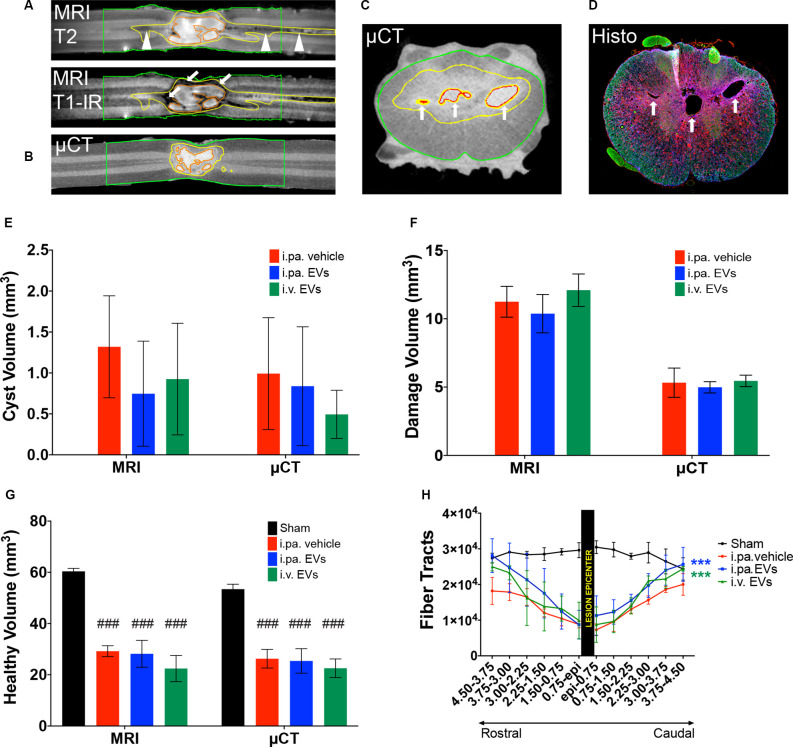
Acute application of EVs improved structural outcomes after tSCI. Representative magnetic resonance (MRI) and micro-computed tomography (μCT) images of vehicle-treated rats at 56 days post-tSCI. Corresponding horizontal sections obtained with **(A)** T1-IR and T2 MRI sequences or **(B)** contrast agent-enhanced μCT. Arrows point to dense scar tissue in **(A)**, arrowheads point to degenerating tracks in **(A)**. Corresponding cross-sections obtained with **(C)** contrast agent-enhanced μCT and **(D)** immunohistology (GFAP in blue, NF-H in green and Iba1 in red). Arrows point to cysts in **(C)** and **(D)**. **(A)** to **(C)**: red lines: cysts; yellow lines: damaged tissue and green lines: sample borders. Quantification of the **(E)** volume of cysts, **(F)** volume of damaged tissue including cysts, and **(G)** volume of intact-appearing tissue. **(E–G)** Statistical differences to sham group using one-way ANOVA and Tukey *post hoc* test on all groups: ^(###)^*p* < 0.001. **(H)** Longitudinal fiber tracts rostral and caudal to the lesion epicenter detected by DTI. Statistical differences to vehicle group using two-way ANOVA and Tukey *post hoc* test on tSCI groups (without sham): ^(***)^*p* < 0.001.

To gain more insight into the morphology of the lesion site at 56 days post-injury, *ex vivo* magnetic resonance imaging (MRI) on the spinal cord was performed before processing the samples for histological analyses. First, T1-IR, T2, and DTI-EPI MRI sequences were acquired (Figures 5A,H), followed by contrast agent-enhanced μCT imaging (Figures 5B,C). MRI and μCT provided a detailed 3D representation of the damaged tissue, revealed the complex cyst morphology, and accurately pinpointed the position of the lesion within the spinal cord (Figures 5C,D). The combination of T1-IR and T2 MRI acquisition sequences was the most sensitive for the detection of altered tissue and allowed differentiating, for example, regions with intensive scarring and those with degeneration of axonal tracts (Figure 5A). MRI and micro-computed tomography (μCT)-based volumetric analyses conducted from 5 mm rostral to 5 mm caudal to the lesion epicenter addressed the total cyst volume (Figure 5E), the total volume of damaged tissue (necrosis, scar, edema, etc.; Figure 5F) and total volume of remaining tissue that appeared to be intact (Figure 5G). Comparison between the various groups did not reveal significant differences between tSCI rats that received EV treatments or vehicle.

*Ex vivo* diffusion tensor imaging (DTI) is a powerful technique to evaluate tissue integrity in the spinal segments surrounding the lesion site. At 56 days post-injury, we examined the integrity of longitudinal fiber tracts using DTI in a 9 mm segment of the spinal cord centered on the lesion. As can be seen in Figure 5H, the number of longitudinal tracts decreased progressively towards the lesion epicenter, reflecting the progressive disappearance of intact axonal bundles. The severity of the lesion precluded a reliable measurement directly at or across the lesion epicenter. Nevertheless, DTI measurements revealed significantly more longitudinal tracts rostral and caudal to the lesion in the injured rats that received the EVs, as compared to the vehicle (*p* < 0.001). Although more fibers were detected following i.pa. application of EVs, as compared to i.v. administration, this difference did not reach statistical significance.

Finally, we assessed whether the number of motor neurons surviving in the ventral horns of the spinal cord correlated with the better motor function recovery observed following i.pa. application of EVs. Immunodetection of choline acetyltransferase (ChAT) expression at 14 days and 56 days post-injury revealed that the number of motor neurons was significantly decreased (approx. 35%) near the lesion epicenter as compared to the sham group. However, no significant differences were detected following the early application of EVs after the injury as compared to the vehicle treatment (Table 2).

## Discussion

To the best of our knowledge, this constitutes the first report showing that an intralesional application of EVs successfully improves long-term structural and functional outcomes in a rat model of tSCI. We have recently published that the potency of intravenous application of the MSC-EVs to diminish inflammation and scarring surpassed the benefits of whole-cell MSC injection in a rat model of tSCI (Romanelli et al., 2019). Here, we demonstrate that an acute application of EVs directly into the lesion site was significantly more potent to improve long-term functional outcomes. From the BBB scores and sub-scores, we propose that the high intralesional concentration of EVs that was obtained by the direct intra-parenchymal application boosts the locomotor recovery after tSCI. Intriguingly, the recovery of normal thermoception after SCI was quicker in rats treated with i.v. EVs than in rats treated with i.pa. EVs. It may be speculated that following intravenous application, EVs have greater access to the soma of neurons located in the dorsal root ganglions which transmit pain and thermal sensations.

The size and position, as well as the complex system of cysts, could be clearly depicted in the spinal cords *en bloc* using contrast agent-enhanced μCT and MRI in rats entering the chronic phase post-tSCI. In contrast, the histological analysis on cryosections was more susceptible to tissue deformation. It should be noted that μCT and MRI revealed the presence of loose undefined material in several cysts that was not detected in the histological analyses. This material, presumably necrotic tissue, may have been too unstable to withstand cryosectioning and histological processing. Using μCT and MRI-based volumetric measurements, we could not detect the slightly larger volume of intact tissue measured by the histological analysis following i.pa. application of EVs. Whether μCT and MRI are less precise for volumetric analyses than histology or whether the small number of spinal cords analyzed causes this discrepancy remains to be clarified.

The preservation and recovery of spinal neuronal network organization is paramount for its functionality. Taken that as little as 7–10% of the neuronal connections across the lesion site may be enough to obtain significant functionality (Eidelberg et al., 1977; Blight, 1983; Fehlings and Tator, 1995; Kakulas, 1999), even small improvements of connectivity could be relevant. We took advantage of the anisotropic water diffusivity in myelinated axonal bundles to perform diffusion tensor imaging (DTI). Due to the small size of the rat spinal cord and the respiratory motion artifacts, we performed our analyses *ex vivo*. The *ex vivo* measurement on the other side probably decreased the detection sensitivity to anisotropy. Nevertheless, at 8 weeks post-tSCI, we detected significantly more fiber tracts along the rostro-caudal axis in rats that had received EV treatments, as compared to the vehicle-treated rats. We hypothesize that the higher fractional anisotropy resulted from a better structural preservation of the white matter tracts. However, in the absence of measurements at earlier time points, we cannot rule out that regenerative processes also contributed to the higher number of fibers detected.

Acute intralesional application of EVs significantly decreased the local expression level of IL-1β and IL-6. Reduction of inflammation during the acute phase post-SCI is thought to improve the long-term functional outcome (Hausmann, 2003; Arnold and Hagg, 2011; Couillard-Despres et al., 2017; Orr and Gensel, 2018). We recently demonstrated that EVs can directly interact with microglia *in vitro* to dampen their inflammatory phenotype (Romanelli et al., 2019; Warnecke et al., 2020). Hence, EVs applied immediately after tSCI likely target resident microglia at the lesion site to reduce the induction of the local inflammatory response. This modulation was still detectable at 14 days post-injury and resulted in significantly reduced inflammation and scarring. Hence, in addition to the reduction in the density of Iba1-expressing cells, EV applications diminished the reactive astrogliosis by roughly 35% at 14 days post-injury. Interestingly, only the intra-parenchymal EV application could significantly reduce the deposition of collagen I and NG2 at the lesion site, which indicates a stronger anti-scarring activity. In contrast to the pathophysiology of tSCI in rodents, astrogliosis and scarring in human tSCI evolve over a longer time span and become prominent at intermediate and chronic stages (Puckett et al., 1997; Hagg and Oudega, 2006). Hence, successful application of EVs against scarring in humans may rely on repeated application, covering a longer time window after tSCI.

The beneficial impact of intravenous application of EVs has been reported using EVs secreted by MSCs of various origins (Huang et al., 2017; Ruppert et al., 2018; Sun et al., 2018; Liu et al., 2019). Although the modes of action of MSC-EVs following tSCI are still to be elucidated, the control of inflammatory processes, reduction of scar formation, and the improvement of vasculature integrity appear to be paramount. Knowledge over the active components of UC-MSC-EVs remains still partial, but the immunomodulatory effects of EVs appear to be relevant to the therapeutic impact. Adenosine signaling may contribute to the immunomodulatory activity of EVs *via* the conversion of AMP to adenosine by the GPI-anchored 5′-ecto-nucleotidase CD73 present on enriched UC-MSC EVs (Priglinger et al., 2021). A recent report by Zhai et al. (2021) demonstrated that the use of EVs produced by virus-transformed hUC-MSC engineered to increase the levels of CD73 alleviated inflammation after SCI in a mouse model, and regulated macrophage polarization *in vitro*. Along the same line, Xu et al. (2018) demonstrated that CD73-deficient mice displayed overwhelming immune responses and poor locomotor recovery after SCI. From these data, the authors concluded that CD73 had a protective effect on secondary damage and that the potential mechanism underlying this effect was the restoration of tissue homeostasis *via* extracellular adenosine signaling.

Recently, an intra-cisternal application of bone marrow derived MSCs was also reported to improve the structural and functional outcomes following tSCI in a rat model (Romero-Ramírez et al., 2020). The biodistribution of the MSC-secreted factors, such as EVs, following intra-cisternal application remains unclear as tSCI provokes swelling of the spinal cord that could hinder cerebrospinal fluid circulation and the distribution of secreted factors. Indeed, the mild impact of MSCs intra-cisternal application reported suggests that these factors may not have reached the lesion epicenter in high concentrations and/or not rapidly enough after tSCI to be as effective as the intralesional application of EVs described in our study.

In conclusion, we have demonstrated that the acute intralesional application of EVs *via* direct intra-parenchymal injection is more effective than the intravenous route to address the early processes of secondary damage in a rat model of tSCI. Furthermore, the intralesional treatment significantly improved the long-term functional outcomes. Even if more invasive in nature than the intravenous application, local injection of biologicals in the spinal parenchyma surrounding the lesion site has been shown to be safe when performed in a slow and gentle fashion in patients with SCI (Levi et al., 2018). Decades of research have indicated that treatment of spinal cord injury will require a manifold approach with different types of intervention for the different phases post-injury. Our observation demonstrates that EVs are particularly potent to address the processes of inflammation and scarring when applied very early and close to the site of injury. Moreover, an improvement of the lesion microenvironment and the additional sparing of parenchyma obtained with EV treatment constitute valuable assets for follow-up interventions aiming for endogenous axonal regeneration or involving cell therapies.

## Data Availability Statement

The original contributions presented in the study are included in the article/Supplementary Material, further inquiries can be directed to the corresponding author.

## Ethics Statement

The animal study was reviewed and approved by Federal Ministry of the Republic of Austria for Education, Science and Research (BMBWF-66.019/0036-V/3b/2018).

## Author Contributions

PR, LB, PH, SŠ, BB, ER, MG, DH, MD, and SC-D designed the project. PR, LB, PH, SŠ, DJ, CK, PZ, TS, MG, DH, and MD performed experimental work and acquired data. PR, LB, DH, MD, and SC-D wrote the manuscript. Manuscript corrections were done by all authors. All authors contributed to the article and approved the submitted version.

## Conflict of Interest

PR was employed by company Innovacell AG. The remaining authors declare that the research was conducted in the absence of any commercial or financial relationships that could be construed as a potential conflict of interest.

## Publisher’s Note

All claims expressed in this article are solely those of the authors and do not necessarily represent those of their affiliated organizations, or those of the publisher, the editors and the reviewers. Any product that may be evaluated in this article, or claim that may be made by its manufacturer, is not guaranteed or endorsed by the publisher.

## Footnotes

^1^ http://dsi-studio.labsolver.org

## Supplementary Materials

Supplementary Figure 1 Bladder functionality after tSCI. Time course of the bladder functionality score after tSCI for vehicle-treated, i.pa. EVs-treated as well as i.v. EVs-treated rats. Urinary bladder voiding function was assessed according to filling volumes detected by palpation (1 = very large; 7 = empty bladder).
