# Supplementary figures and images for: Enhancing Functional Recovery Through Intralesional Application of Extracellular Vesicles in a Rat Model of Traumatic Spinal Cord Injury
Source: Front Cell Neurosci. 2022 Jan 3;15:795008. doi: 10.3389/fncel.2021.795008 (PMC8762366; doi:10.3389/fncel.2021.795008)

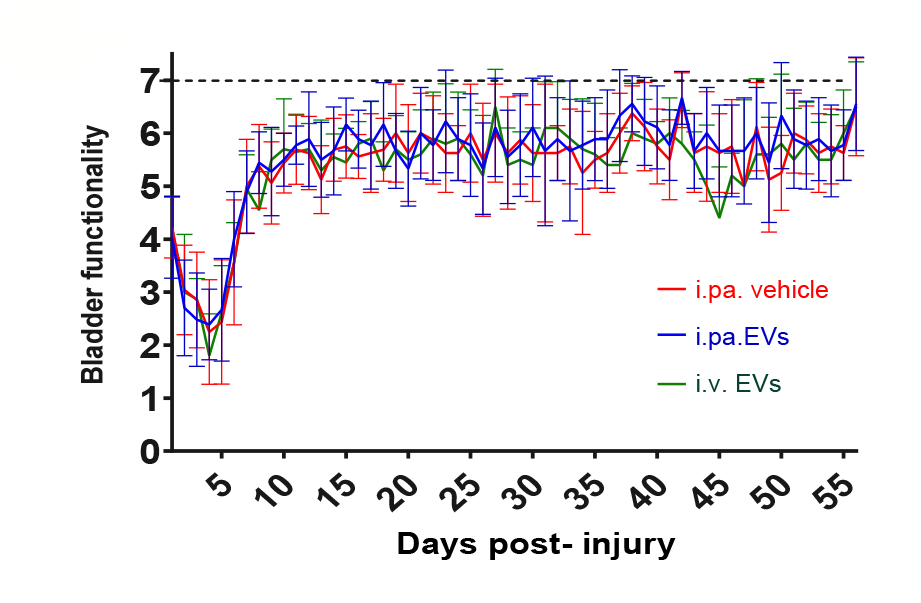

Supplement: Supplementary Figure 1 — Bladder functionality after tSCI. Time course of the bladder functionality score after tSCI for vehicle-treated, i.pa. EVs-treated as well as i.v. EVs-treated rats. Urinary bladder voiding function was assessed according to filling volumes detected by palpation (1 = very large; 7 = empty bladder). [file Image_1.TIF]
